# Hemifacial spasms triggered by compression of tortuous anterior inferior cerebellar artery loop on the facial nerve in the internal auditory canal: A case report

**DOI:** 10.1097/MD.0000000000039690

**Published:** 2024-09-13

**Authors:** Shuide Chen, Siyuan Pan, Seidu A. Richard, Zhigang Lan

**Affiliations:** a Department of Neurosurgery, West China Xiamen Hospital, Sichuan University, Xiamen, P. R. China; b Department of Neurosurgery, West China Hospital, Sichuan University, Chengdu, Sichuan, P. R. China; c Institute of Neuroscience, Third Affiliated Hospital, Zhengzhou University, Zhengzhou, P. R. China.

**Keywords:** AICA loop, facial nerve, HFS, IAC, MVD, tortuous

## Abstract

**Rationale:**

Hemifacial spasm (HFS) is triggered by neurovascular compression mostly at the root entry/exit zone of the facial nerve. HFS with the responsible blood vessel located in the internal auditory canal (IAC) is a very rare occurrence. In our case, the HFS was triggered by compression of the anterior inferior cerebellar artery (AICA) loop on the facial nerve in the IAC.

**Patient concerns:**

A 27-year-old female presented with a 5-year history of right-sided facial twitching with no obvious course. The frequency and severity of the attacks increases when the patient was anxious or agitated which severely affected her quality of life.

**Diagnosis:**

Preoperative 3D-TOF magnetic resonance imaging (MRI) evaluation of cranial nerves showed that the right AICA loop had a tortuous course within the IAC and compressed the facial nerve.

**Intervention:**

Microvascular decompression (MVD) surgery was carried out to separate the tortuous AICA loop and facial nerve in the IAC using a Teflon pad.

**Outcomes:**

The abnormal muscle response disappeared intraoperatively and 2-years follow-up revealed no recurrence of her symptomatology. She is current well and go about her daily activities with no neurological deficits.

**Lesson:**

The attachment of the facial nerve to the tortuous AICA loop coupled with the pulsatile impulse of tortuous AICA loop may have resulted in the entrapment and compression of the CN VII in the IAC.

## 1. Introduction

Hemifacial spasm (HFS) is triggered by neurovascular compression mostly at the root entry/exit zone (REZ) of the facial nerve (CN VII).^[1,2]^ HFS is depicted with involuntary and intermittent ipsilateral eyelid twitching with both tonic as well as spastic movements, typically advancing to all ipsilateral facial muscles, leading to facial disfigurement.^[2]^ Anatomically, the anterior inferior cerebellar artery (AICA) loop denotes an anomalous course of the AICA that enters the internal auditory canal (IAC) or meatus and loops over the CN VII and auditory nerve (CN VIII).^[3]^ In some cases, this was associated with facial, audiological and vestibular symptoms as a result of vascular compression.^[3]^

Also, the AICA courses backward around the pons 3-dimensionally (3D) toward cerebellopontine angle (CPA) cistern to supply the inferolateral pons, middle cerebellar peduncle, flocculus, as well as the anterior margins of cerebellar hemispheres.^[4]^ Magnetic resonance imaging (MRI) with high-resolution such as thin-slice sequences through the CPA (MR cisternography) combined or fused with 3D time-of-flight spoiled gradient-recalled angiography sequence (3D-TOF MRA) allows for effective diagnosis as well as evaluation of neurovascular compression and conflicts.^[5]^ Microvascular decompression (MVD) surgery is the utmost effective treatment modality for HFS because, it absolutely relief the symptomatology compared with treatment modalities like medication and botulinum toxin.^[2,6,7]^

Notably, the efficacy of MVD surgery for HFS is closely related to the identification and decompression of the responsible blood vessels during surgery. The overall symptom free rate after MVD surgeries for HFS was about 90%, with the remaining 10% of HFS surgeries associated with recurrence due to failed surgery.^[7]^ Interestingly, HFS with the responsible blood vessel located in the IAC is a very rare occurrence. We report a case of HFS triggered by compression of the AICA loop on the CN VII in the IAC who was effectively manage via MVD with no recurrence.

## 2. Case report

A 27-year-old female presented with a 5-year history of right-sided facial twitching with no obvious course. The facial twitching was initial mild but became severe 3 years prior to admission. The facial twitching was mainly observed as involuntary intermittent twitching at the right eyelid during eye blinking and extended to the right side of the month other over last 3 years prior to admission. The frequency and severity of the attacks increases when the patient was anxious or agitated which severely affected her quality of life. She denied history of trauma. She also denied symptoms such as headache, dizziness, facial asymmetry, incomplete eye closure, choking while drinking water, or weakness in the limbs.

On physical examination, she was alert and her face articulated symmetrically with equally sized pupils that were sensitive to light. Also, her forehead wrinkled symmetrically and her nasolabial folds were symmetrical. Her right eyelid and mouth were involuntary twitching during eye blinking and mouth opening respectively. Her tongue was centered with no deviations on protrusion. Also, the neck was soft and the muscle strength as well as the tone of her limbs were normal. Rinne and Webber hearing tests were both normal. All routine laboratory investigations, Chest X-ray and electrocardiogram were normal.

**Figure 1.**
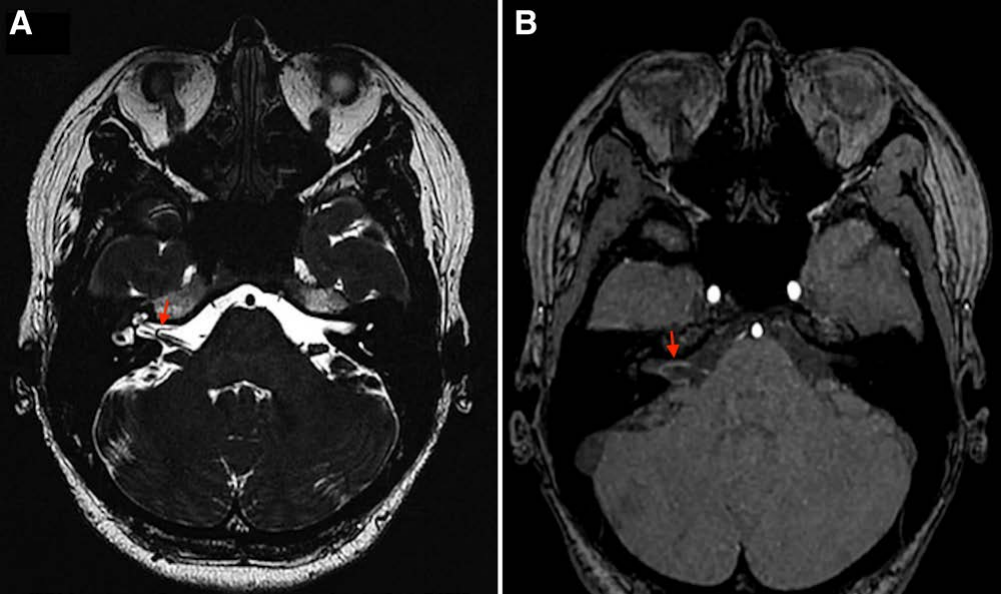
(A and B) Are 3D-TOF MRI showed that the right CN VII penetrated into the IAC and was closely related to the AICA loops (red arrows).

Preoperative 3D-TOF MRI evaluation of cranial nerves showed that the right AICA had a tortuous course around its loop point within the IAC. Interestingly, the AICA loop was closely compressing the adjacent CN VII and CN VIII in the IAC [Fig. 1(A) and (B)]. Preoperative neurophysiological evaluation revealed the presence of lateral spread response on the right side of the face. Based on the MRI finding above, a diagnosis of HFS cause by the compressive effect of the right AICA loop on the CN VII in the IAC was made. Thus, the patient was scheduled for CN VII MVD surgery.

**Figure 2.**
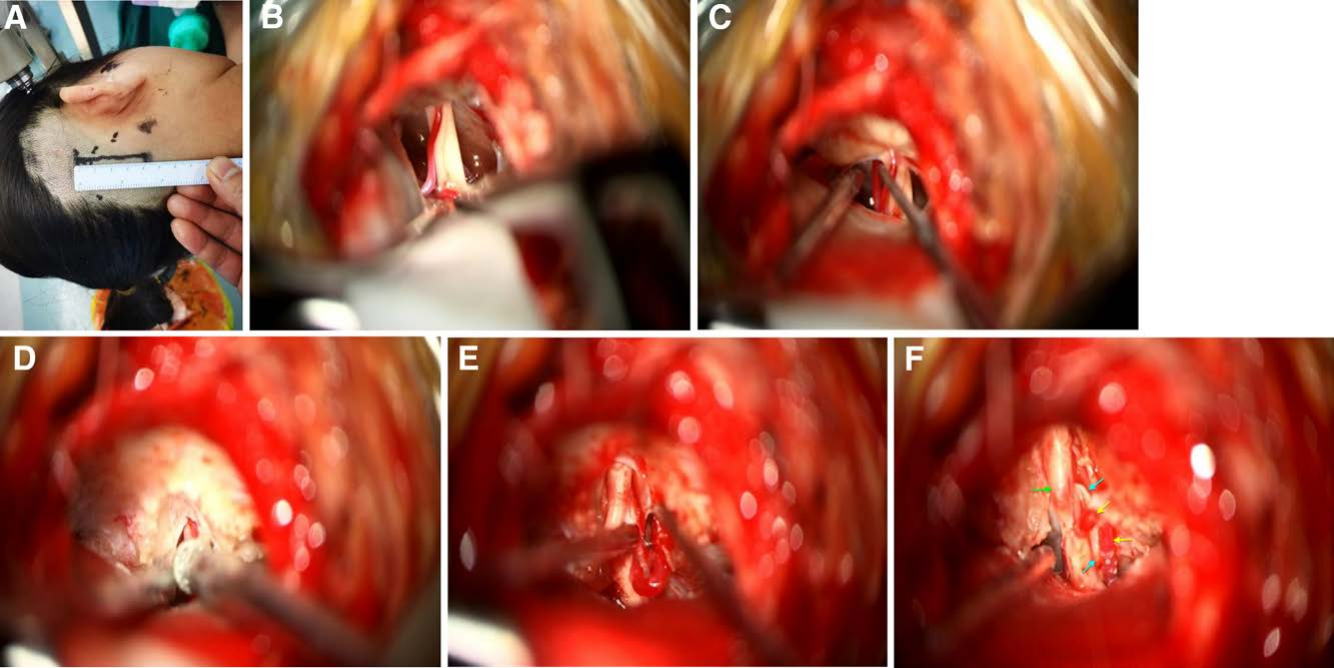
(A–F) Are intraoperative images show the sequence of the operation modality. (A) Show the marking of the incision used to access the lesion at the CPA. (B and C) Show that there was no vascular entrapment of the CN VII at the REZ, and the CN VII and CN VIII were accompanied by the AICA which all penetrated deep into the IAC. (D) Show micro-drilling of IAC to expose the AICA loop and the CN VII. (E) Show the exposed the AICA loop and the CN VII. (F) Shows the AICA loop (yellow arrow), the Teflon pad (blue arrow) and the CN VII (green arrow).

The patient was put on the park bench position with her head fixed in Mayfield 3 keys’ head support system after general anesthesia. Electromyographic (EMG) and auditory brainstem responses were used to monitor the cranial nerves. The retrosigmoid approach [Fig. 2(A)] was used to access the responsive cranial nerves and the AICA loop in the IAC. Intraoperative exploration revealed that from both REZs of the CN VII and CN VIII, the right AICA running between the CN VII and CN VIII deep into the IAC [Fig. 2(B) and (C)]. Micro-drilling of the IAC was done [Fig. 2(D)] and a tortuous right AICA loop that penetrated 15 mm into the IAC was fully exposed [Fig. 2(E)]. The right AICA was closely adhering to the CN VII. Thus, the responsible blood vessel was the tortuous right AICA loop. We did not observer any other vessels at the REZs of both nerves and the AICA looping was type III.

After carefully dissecting the arachnoid adhesions between the CN VII and CN VIII, the 2 nerves were fully separated and a Teflon pad was used to separate the right tortuous AICA loop and the CN VII [Fig. 2(F)]. The Teflon pad was properly fixed to adjacent dura outside the IAC to prevent the loop from falling back into the IAC with 9-0 sutures. Interestingly, intraoperative neurophysiological monitoring showed that the lateral spread abnormal muscle response (AMR) disappeared. Watertight closure of the dura was achieved and the removed bone flap was replaced and fixed to skull with titanium connecting pieces and titanium nails. The skin was closed afterword.

**Figure 3.**
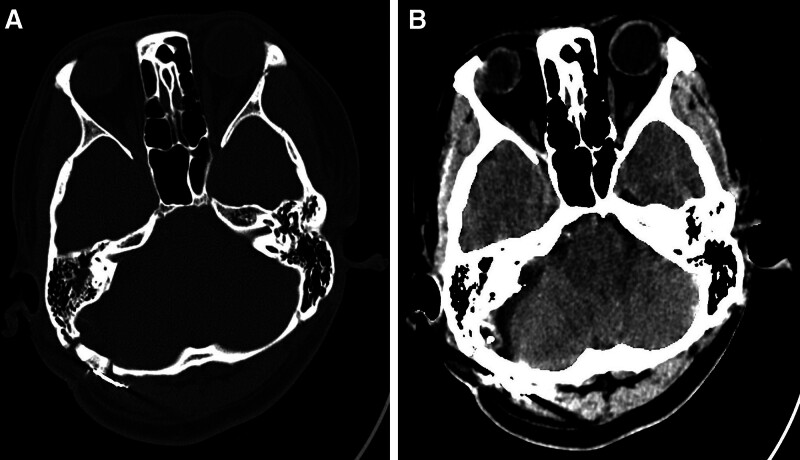
(A and B) Are postoperative computer tomography (CT) scan showing no bleeding or infarction.

Her HFS completely disappeared after awakening from anesthesia. Moreover, there were no complications such as facial paralysis, hearing loss, tinnitus, cerebrospinal fluid (CSF) leakage, or subcutaneous fluid accumulation. A postoperative computer tomography scan done on the same day showed no bleeding or infarction [Fig. 3(A) and (B)]. She was discharged home 7 days later and 2-years follow-up revealed no recurrence of her symptomatology. She is current well and go about her daily activities with no neurological deficits.

## 3. Discussion

The most common etiology of HFS is compression of the CN VII that exits the brainstem by an anomalies or aberrant artery.^[8]^ Specifically, aberrant arterial anomalies in the branches of the AICA, posterior inferior cerebellar artery, and vestibular artery often compress the CN VII root.^[8]^ Our patients HFS was triggered by compression of the tortuous AICA loop on the CN VII.^[8]^ Anatomically, AICA loops are classified into type I to III.^[3]^ Type I denotes the lying of the AICA loops only in the CPA, but not entering the IAC, type II denotes the entering AICA loops into the IAC, but not extending more than 50% of the length of the IAC while type III denotes the entering AICA loops into the IAC and extending more than 50% of the length of the IAC.^[3]^

Contrarily, some studies have shown that AICA loops themselves are not pathological but are normal anatomical structures that are located at the IAC.^[4,9]^ Notably, the REZ of CN VII at the brainstem lacks Schwann cell wrapping and is highly sensitive to pulsatile as well as vascular compression. The most common responsible vessel for CN II compression is the AICA.^[2,10]^ Also, tortuous vessels can occur at the REZ, CPA, as well as the IAC. In our case, the attachment of the CN VII to the tortuous AICA loop coupled with pulsatile impulse of tortuous AICA loop may have resulted in the entrapment and compression of the CN VII in the IAC.

HFS is usually characterized with paroxysmal recurrent involuntary twitching of muscles of facial expression and other muscles innervated by the CN VII.^[8,11]^ This symptom become aggravated when the patient is excited or anxious.^[11]^ Notably, the clinical presentation in our case was same as above. Also, vascular compression from AICA has not been found to be a causative factor for tinnitus.^[12]^ Similarly, the AICA classification was not associated with otoneurologic symptomatology.^[13]^ Clinical evaluation of our patient did not indicate the involvement of otoneurological sequalae.

The common sequences for the evaluation of cranial nerves includes MRI with 3D-CISS, 3D-TOF and 3D-FIESTA-C.^[5,14]^ In our case, preoperative 3D-TOF MRI sequences preliminarily determined that the right AICA loop on the affected side had a tortuous course which was associated with the compression of CN VII at the IAC.

MVD is the utmost effective treatment modality for HFS.^[2,6,7]^ Identifying the responsible vessel and separating it from the nerve is very critical during MVD surgery.

However, postoperative recurrence of HFS after MVD surgeries are mainly related to improper placement and displacement or detachment of the Teflon pad causing the responsible vessel to return to its original position.^[15]^ Thus, the pulsatile impulse of the overly thin responsible vessel continues to transmitted to the CN VII leading to recurrence.^[15]^ Also, postoperative arachnoid adhesions and granuloma formation around Teflon pad have been implicated in recurrences.^[15–24]^

Ama-Gasaki et al observed a 90% cure rate of HFS using a suspension method which involved fixing the responsible vessel to the cerebellum or petrous bone with a Teflon pad.^[6]^ Intraoperatively, we carried out micro-drilling of the IAC to fully exposed the tortuous AICA loop that penetrated 15mm into the IAC and was closely adhering to the CN VII. Also, we used a Teflon pad to separate the tortuous AICA loop and properly suspended and fixed it on the petrous dura mater with 9-0 sutures to prevent the displacement.

Interestingly, the AMR disappeared immediately, indicating that the tortuous AICA loop was completed separated from the CN VII. We did not observe complications such as facial paralysis, hearing loss, tinnitus, CSF leakage, or subcutaneous fluid accumulation. Also, 2-years follow-up revealed no recurrence of her symptomatology. She is current well and go about her daily activities with no neurological deficits.

## 4. Conclusion

The attachment of the CN VII to the tortuous AICA loop coupled with the pulsatile impulse of tortuous AICA loop may have resulted in the entrapment and compression of the CN VII in the IAC. Also, identifying the responsible vessel and separating it from the nerve is very critical during MVD surgery. Moreover, intraoperative EMG and ABR monitoring of the cranial nerves is very critical during MVD surgery.

## Author contributions

**Conceptualization:** Shuide Chen, Siyuan Pan, Seidu A. Richard, Zhigang Lan.

**Data curation:** Shuide Chen, Siyuan Pan, Seidu A. Richard, Zhigang Lan.

**Formal analysis:** Shuide Chen, Siyuan Pan, Seidu A. Richard, Zhigang Lan.

**Investigation:** Shuide Chen, Zhigang Lan.

**Methodology:** Shuide Chen, Siyuan Pan, Seidu A. Richard, Zhigang Lan.

**Resources:** Zhigang Lan.

**Supervision:** Zhigang Lan.

**Writing – original draft:** Shuide Chen, Seidu A Richard.

**Writing – review & editing:** Shuide Chen, Siyuan Pan, Seidu A. Richard, Zhigang Lan.
